# Engineering a Circular Riboregulator in *Escherichia coli*

**DOI:** 10.34133/2020/1916789

**Published:** 2020-09-12

**Authors:** William Rostain, Shensi Shen, Teresa Cordero, Guillermo Rodrigo, Alfonso Jaramillo

**Affiliations:** ^1^Warwick Integrative Synthetic Biology Centre (WISB) and School of Life Sciences, University of Warwick, CV4 7AL Coventry, UK; ^2^Institute of Systems and Synthetic Biology, CNRS-Université d’Évry Val-d’Essonne, 91000 Évry, France; ^3^Instituto de Biología Molecular y Celular de Plantas, CSIC-Universidad Politécnica de Valencia, 46022 Valencia, Spain; ^4^Institute for Integrative Systems Biology (I2SysBio), CSIC-Universitat de València, 46980 Paterna, Spain

## Abstract

RNAs of different shapes and sizes, natural or synthetic, can regulate gene expression in prokaryotes and eukaryotes. Circular RNAs have recently appeared to be more widespread than previously thought, but their role in prokaryotes remains elusive. Here, by inserting a riboregulatory sequence within a group I permuted intron-exon ribozyme, we created a small noncoding RNA that self-splices to produce a circular riboregulator in *Escherichia coli*. We showed that the resulting riboregulator can *trans*-activate gene expression by interacting with a *cis*-repressed messenger RNA. We characterized the system with a fluorescent reporter and with an antibiotic resistance marker, and we modeled this novel posttranscriptional mechanism. This first reported example of a circular RNA regulating gene expression in *E. coli* adds to an increasing repertoire of RNA synthetic biology parts, and it highlights that topological molecules can play a role in the case of prokaryotic regulation.

## 1. Introduction

RNA molecules can regulate gene expression in *trans* through many different mechanisms in bacteria, either by themselves such as with positive/negative control of protein translation [1–3] and transcription termination [4, 5] or in complex with proteins such as in CRISPR interference [6]. These developments have allowed the application of small RNAs (sRNAs) to engineer synthetic circuits that can be used for sensing, computation, and control. Examples include circuits that can count [7], paper-based sensors for programmable *in vitro* diagnostics [8], systems for *in vivo* RNA structure determination [9], or microorganisms with robust controlled growth [10]. In this work, we focus on riboregulators, *trans*-regulating sRNAs that interact with *cis*-repressed messenger RNAs (mRNAs) to activate protein translation [1], which are easily programmable as their interactions are mostly governed by simple base pairing rules. So far, natural and synthetic riboregulators used in bacterial cells have been linear. Here, we propose to explore the possibility of engineering a circular riboregulator, which would highlight a novel posttranscriptional event of control in bacteria. Of note, our aim is not to show that circular riboregulators could perform better than linear ones, but to demonstrate that topological molecules can regulate gene expression even in simple organisms like bacteria by engineering one instance.

Circular RNAs were long considered oddities, and it has only recently become clear that they are widespread regulators of gene expression in some organisms [11–13]. The development of methods such as CircleSeq [11] has shown that circular RNAs are abundant and important regulators in animals [12, 13], where they have been found to act as high-stability sponges that sequester and inactivate microRNAs. However, to our knowledge, no circular RNA has been found or engineered to directly interact with the mRNA to control gene expression. Our proposal addresses this issue, exploring if bacterial cells can accommodate circular sRNAs interacting with mRNAs.

One way of creating artificially circularized RNAs [14, 15] is to exploit group I ribozymes [16], using the permuted intron-exon (PIE) method [17, 18]. PIE ribozymes self-splice through two sequential *trans-*esterifications, ligating the two exons, and require only GTP and Mg^2+^ for activity. A rearrangement of the order of the introns and exons at the DNA level, by removing the dispensable P6 loop (Figure 1(a)), results in the circularization at the RNA level of the sequence inserted between the two exons (Figures 1(b) and 1(c)) [14, 18]. In this work, we exploit this mechanism to design a ribozyme (using the sequence from phage T4) that yields a circular molecule that contains a given sRNA fragment appropriately inserted (Figure 1(c)). We design an sRNA sequence so that the resulting structure of the circular molecule (accounting for the exons) does not interfere with its regulatory ability. We prove experimentally that the produced molecule is indeed circular and that it can regulate gene expression in *Escherichia coli*. For that, we follow a simple strategy by which the 5${}^{\prime }$ untranslated region (5${}^{\prime }$ UTR) of the mRNA *cis*-represses the ribosome binding site (RBS). When present, the circular riboregulator interacts with the 5${}^{\prime }$ UTR, displacing the stem, exposing the RBS, and activating protein translation (Figure 1(d)) [1].

Figure 1Diagram of group I and PIE ribozymes [14, 18] and mechanism of riboregulation. (a) Left: secondary structure of wild-type group I intron, with the dispensable P6 loop colored in green, introns in pink, and exons in blue. Right: structure of the PIE ribozyme, with riboregulator RAJ31 [19] inserted between the exons. (b) Sequence scheme in the wild-type group I ribozyme and illustration of products after self-splicing. (c) Sequence scheme in the PIE ribozyme, where the order of the introns and exons is inverted, and illustration of products after self-splicing, including the circularized sRNA. (d) Diagram of riboregulation with a circular sRNA. A *cis*-repressed mRNA is *trans*-activated by the circular sRNA, which induces a conformational change in the 5${}^{\prime }$ UTR to release the RBS and then allow translation initiation (see sequences and structures of our design in Figures S1 and S2).

**(a):**
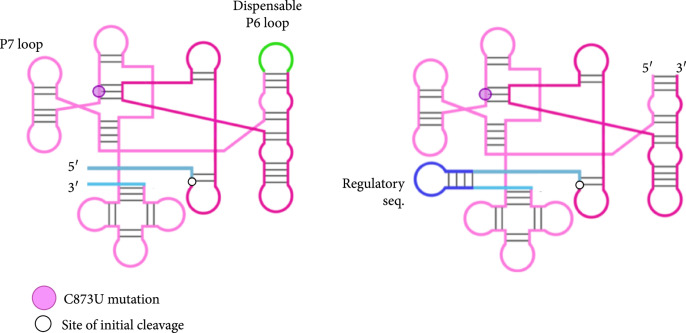

**(b):**
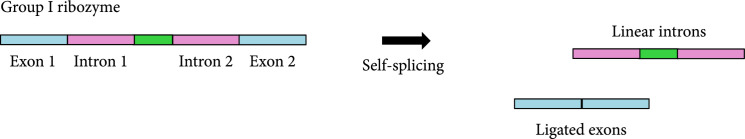

**(c):**
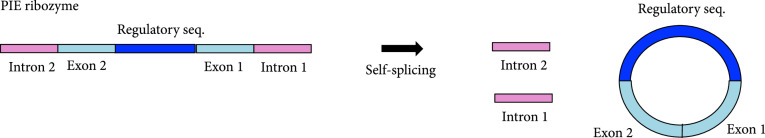

**(d):**
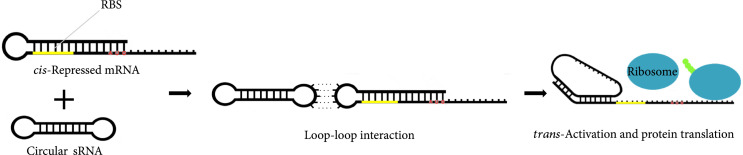

## 2. Materials and Methods

### 2.1. Plasmid Construction

All cloning was performed using *E. coli* DH5*α* cells (Life Tech). Riboregulator circRAJ31 (Table S1) was synthesized by IDT in plasmid pIDTSmart (ampicillin resistance, pMB1 origin of replication with a high copy number). The mRNAs cisRAJ31-GFP, cisRAJ31-GFP-LAA, and cisRAJ31-cat were expressed from plasmid pRAJ31m [19], a pSB4K5-derived plasmid (kanamycin resistance, pSC101-derived origin of replication, with mutation E93G in *repA* [20], with a medium copy number). The *trans*-activating sRNA circRAJ31 and the C873U, G423A, and toehold mutants (Table S1), as well as the *cis*-repressed mRNA cisRAJ31-cat, were constructed by PCR using appropriate primers (Table S2). A linear version of circRAJ31 was also constructed (removing the introns and exons, named RAJ31min). Constructs were cloned into pIDTSmart (for sRNAs) or pSB4K5-derived (for mRNAs) plasmids. A construct with a T7 polymerase promoter was used to express *in vitro* circRAJ31 and the C873U mutant from plasmid pSB1C3 (chloramphenicol resistance, pMB1 origin of replication). Plasmids were sequenced (GATC Biotech) using primer M13-Fw/M13-Rv (pIDTSmart plasmids) or VF2/VR (pSB4K5-derived plasmids). Unless otherwise noted, all primers were purchased from IDT and chemicals from Sigma-Aldrich.

### 2.2. RT-PCR Assays

RNA was produced in *E. coli* BL21(DE3) cells. Three colonies of each sample were grown overnight in LB medium, then refreshed at 1 : 200 in 20 mL, grown for 4 h in the presence of 125 *μ*M IPTG and 35 *μ*g/mL chloramphenicol, spun down, and resuspended in 400 *μ*L TE buffer. 400 *μ*L cold methanol was added, and cells were disrupted with 0.1 *μ*m beads, followed by acidic phenol-chloroform extraction (Ambion) and ethanol precipitation with 50 *μ*g/mL glycogen (Ambion) and 0.3 M sodium acetate and then resuspended in water. RNA was treated with DNase I (NEB), and acidic phenol-chloroform extraction was repeated. For *in vitro* transcribed RNA, a TranscriptAid T7 high-yield transcription kit (Life Tech) was used following the manufacturer’s instructions. Reverse transcription polymerase chain reaction (RT-PCR) was performed using GoTaq Green MM (Promega). Primer-specific RT was performed using circRT-Rv and M-MuLV-Reverse Transcriptase (Life Tech). Cycling conditions for PCR were as follows: 2 min at 95°C, then 35 cycles of 15 s at 95°C, 30 s at 58°C, and 30 s at 72°C, and finally 5 min at 72°C. Primers (circRT-Fw and circRT-Rv for detecting a circular product; circRT-Rv and circRT-Lin for an unspliced product) were used at 150 nM. Products were then run on a 2% gel for 1.5 h at 85 V and sequenced using primer circRT-Rv after purification.

### 2.3. Electrophoresis Assays

Two-dimensional (2D) urea-polyacrylamide gel electrophoresis (urea-PAGE) was run with RNA extracted as above. The first dimension was run in 5% polyacrylamide, the second dimension in 7.5% polyacrylamide, and both in TBE buffer with 8 M urea for 1.5 h at 200 V. 2D electrophoresis begins with an electrophoresis in the first dimension to separate the RNA molecules according to their mass. The gel is then rotated 90 degrees, so the molecules are separated again in the second dimension. Circular RNAs can then be identified because they lie out of the diagonal in the gel. This is because topological species migrate in a manner different than that of linear species. For northern blot, the RNA gel was electroblotted to a nylon membrane with positive charge and then immobilized through covalent linkage by UV light. The hybridization was done with 106 counts/min of ^32^P-labelled circRAJ31 of complementary polarity. Band intensities were quantified with ImageJ2 [21].

### 2.4. Fluorescence Assays

For fluorescence assays, plasmids were cotransformed in *E. coli* DH5*α*Z1 cells [22]. Plates and liquid cultures contained ampicillin, kanamycin, and spectinomycin to maintain plasmids and the Z1 cassette. M9 medium used contained 1x M9 salts, 0.1 mM CaCl_2_, 2 mM MgSO_4_, 10 *μ*M FeSO_4_, 0.8% glycerol, 0.2% casamino acids, 1 *μ*g/mL thiamine, 20 *μ*g/mL uracil, and 30 *μ*g/mL leucine and was adjusted to pH 7.4 with NaOH. Three single colonies were grown overnight in LB medium. The next morning, for each condition, 1 mL of M9 medium was inoculated with 5 *μ*L of overnight culture and then grown for 6 h with shaking at 37°C. 5 *μ*L of refreshed culture was then used to inoculate 195 *μ*L of M9 medium with antibiotics and appropriate inducers (100 ng/mL aTc and 250 *μ*M IPTG, unless specified otherwise). Three technical replicates were grown for each sample. Fluorescence measurements were then made in a TECAN Infinite F-500 fluorometer, with 4 cycles/h of shaking with incubation at 37°C followed by measurement of OD_600_ and fluorescence (excitation 465/35 nm, emission 530/25 nm). For each sample, fluorescence over OD_600_ after background subtraction (corresponding to M9 medium) was taken for the time points closest to $\text{O}{\text{D}}_{600}=0.5$ [23]. Autofluorescence of plain cells was then subtracted, unless otherwise specified. Error bars in the figures correspond to the standard deviations between three biological replicates.

### 2.5. Antibiotic Resistance Assays

For activation of chloramphenicol resistance assays, three single colonies of cotransformed *E. coli* DH5*α*Z1 cells were picked and grown overnight in LB. In the morning, they were diluted to 1 : 1000 in LB, grown for 2 h, serially diluted, and plated on various chemicals. All plates contained 35 *μ*g/mL kanamycin, 100 *μ*g/mL ampicillin, and 100 *μ*g/mL spectinomycin, as well as 35 *μ*g/mL chloramphenicol, 100 ng/mL aTc, and 250 *μ*M IPTG if appropriate. Plates were then incubated for 20 h at 37°C, and colonies were counted. For liquid cultures, cells were grown as for fluorescence measurements, refreshed in M9 medium without inducers, and then inoculated into 96-well plates with chemicals as appropriate (70 *μ*g/mL chloramphenicol, 100 ng/mL aTc, and 125 *μ*M IPTG, unless otherwise specified).

## 3. Results

### 3.1. Producing a Circular Riboregulator in *E. coli*

To design the sequence of our circular riboregulator (which we name here circRAJ31), we took a fragment of our previously engineered sRNA RAJ31 (RAJ31min) [19]. This fragment contains the full intermolecular hybridization region with the cognate 5${}^{\prime }$ UTR. We selected this sRNA because it is composed of a stem and a large loop, and its toehold (the sequence triggering the RNA-RNA interaction) is within the loop [24]. The predicted secondary structure of circRAJ31 (after splicing and circularization) still exposes the toehold to the solvent (Figure S1; see also Figure S2 for the secondary structure of the 5${}^{\prime }$ UTR). The sequences of RAJ31min and circRAJ31 are shown in Table S1. To verify that the engineered T4-PIE ribozyme retains its self-splicing ability after insertion of RAJ31min in between the exons, we first performed an RT-PCR, with either convergent or divergent primers [11], using as a template purified RNA from *E. coli* cells expressing the sRNA circRAJ31. Convergent primers amplify the unspliced product, whilst divergent primers amplify the circular spliced product if it exists (Figures 2(a) and 2(b)). The divergent primers hybridize (back to back) in the riboregulator region. The convergent primer pair is formed of one of those, combined with another hybridization in the intron 2 region. Following this simple strategy, the unspliced and circular products were detected in cells containing the sRNA circRAJ31 (Figure 2(c); note that the size of the mature circular molecule is 111 nucleotides), showing that not all sRNAs are circularized and a fraction of linear sRNA remains. Sequencing of the RT-PCR product showed that the ligation of the two exons happens at the expected site (Figure 2(d)). Moreover, a 2D urea-PAGE [11], with a linear probe complementary to the sRNA, further confirmed the circular topology of the sRNA circRAJ31 (Figures 2(e) and 2(f)). The gel also revealed the linear products that are unspliced, partly spliced, and fully spliced. This was again performed with purified RNA from *E. coli* cells expressing the sRNA circRAJ31 [23]. We quantified the products to find that the fraction of fully spliced sRNA (circular or linear molecule of 111 nucleotides) with respect to total sRNA transcribed is 28%. Moreover, once the sRNA is fully spliced, 67% is circularized. This gives a global efficiency of circularization of 18%. Taken together, our results show that insertion of RAJ31min within the T4-PIE scaffold yields a circular sRNA *in vivo.*

Figure 2Detection of the engineered circular riboregulator *in vitro*. (a) Sequence scheme of the riboregulator encased in the PIE ribozyme, which produces a circular RNA molecule after self-splicing. Left: diagram of RT over a circular RNA template, showing the creation of a cDNA with multiple copies of the riboregulator due to a rolling cycle, until the RNA is cleaved by the RNase H subunit of the transcriptase. Right: diagram of RT over a linear RNA template, showing the creation of a cDNA with a single copy of the riboregulator. The RT is initiated with primer circRT-Rv (labelled B). (b) Diagram of PCR over the nascent cDNA with appropriate primers. On the one side, PCR with divergent primers (circRT-Fw in red, labelled A, and circRT-Rv in black) only yields a product if the RNA template is circular. On the other hand, PCR with convergent primers (circRT-Lin in blue, labelled C, and circRT-Rv in black) only yields a product if the RNA template includes intron 2 (i.e., if the self-splicing has not occurred, and then, the RNA template is linear). Note that primers A and B hybridize in the riboregulator region, whilst primer C does in the intron 2 region. (c) Agarose gel of RT-PCR with primers described above of total RNA extracted from *E. coli* cells expressing circRAJ31 or the C873U mutant. The first lane corresponds to Invitrogen 100 bp DNA ladder. Lanes labelled 1 and 2 correspond to duplicates of the RT reaction. The last lane corresponds to PCR without doing RT (control). Top: there is a product in the case of both circRAJ31 and C873U mutant with primers B and C, indicating that there is always a fraction of noncircularized RNA. Bottom: there is a clear product in the case of circRAJ31 with primers A and B, indicating that circularization has taken place. In the case of the C873U mutant, the product is difficult to see. (d) Sequencing chromatogram of the RT-PCR product (with primer B) from circRAJ31 produced *in vivo*. This shows the alignment between the theoretical circular RNA sequence and the actual sequence. The sequencing result covering exons 1 and 2 shows that the splicing and ligation happen at the expected site. See also the gel and sequencing result of RT-PCR from RNA produced *in vitro* with T7 polymerase in Figure S3. (e) Urea gel of total RNA extracted from *E. coli* cells expressing circRAJ31. The first lane corresponds to Thermo Sci. RiboRuler low-range 100 bp RNA ladder. Other lanes correspond to extractions in triplicate from different colonies. A lane marked with the asterisk was used for subsequent 2D analysis. (f) On the top, 2D urea-PAGE (here, 1D: 5%, 2D: 7.5% polyacrylamide) of total RNA extracted from *E. coli* cells expressing circRAJ31. On the bottom, radioactive hybridization with a probe targeting circRAJ31. Linear RNAs show along a diagonal in 2D, whilst circular RNAs show off this diagonal.

**(a):**
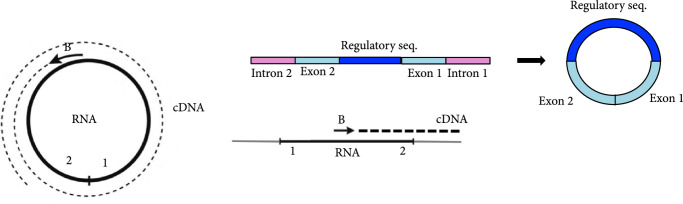

**(b):**
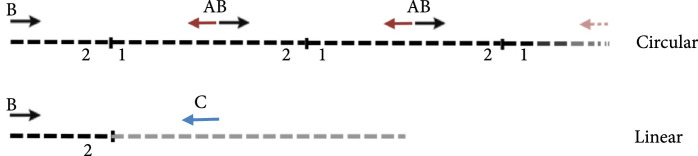

**(c):**
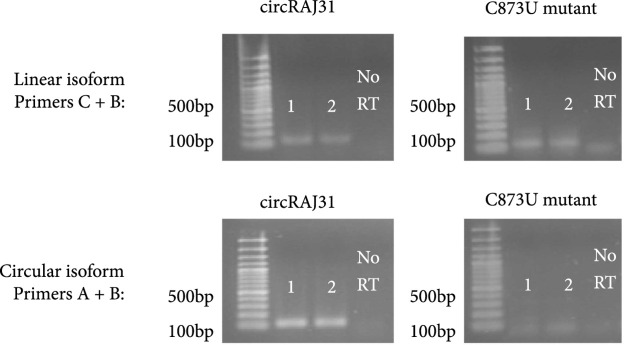

**(d):**
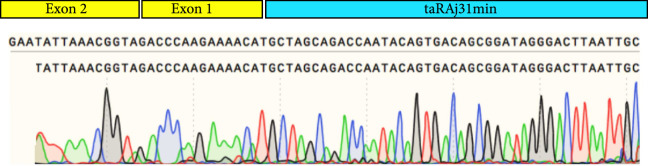

**(e):**
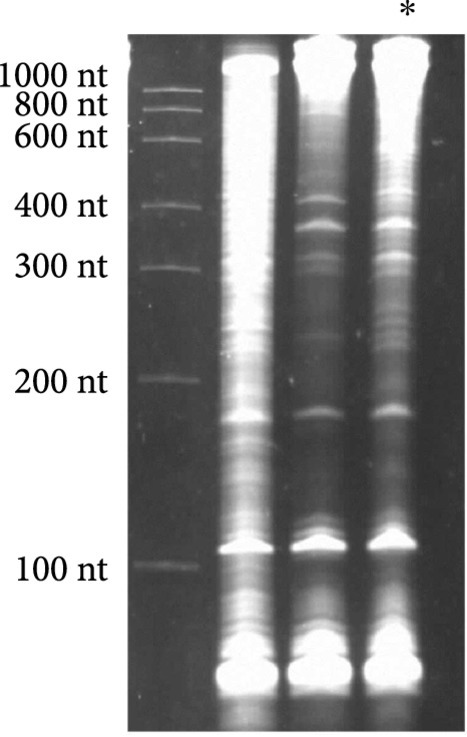

**(f):**
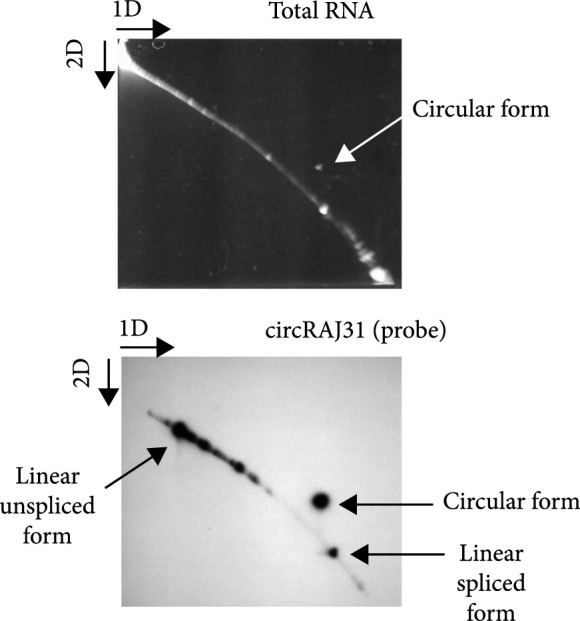

### 3.2. Controlling Protein Translation with a Circular Riboregulator

We then characterized the activity of the circular riboregulator on the expression of a *cis*-repressed reporter gene in *E. coli*. We placed the sRNA circRAJ31 under the control of the inducible promoter P_LtetO1_ [22] on one plasmid and the cognate 5${}^{\prime }$ UTR with the reporter gene (mRNA) under the control of the inducible promoter P_LlacO1_ [22] on another (Figure 3(a)). We used a superfolder green fluorescent protein (sfGFP) with a degradation tag (LAA) as a reporter. We cotransformed the plasmids into *E. coli* DH5*α*Z1 cells, which overexpress the LacI and TetR repressors, providing a tight control of transcription with isopropyl-*β*-D-thiogalactopyranoside (IPTG) and anhydrotetracycline (aTc), respectively. High fluorescence was observed only in the presence of both IPTG and aTc, showing that the riboregulator circularized by the T4-PIE ribozyme can activate gene expression (Figure 3(c)). We also performed an analysis of dose-response of varying inducer concentrations (Figure S5) to show how the system fine-tunes the output expression level. To prove that the RNA-RNA interaction between the circular riboregulator and the 5${}^{\prime }$ UTR of the mRNA is produced as designed, we constructed a toehold mutant where four bases were mutated within the toehold of circRAJ31 (Figure 3(b)). Toehold mutants are important control systems to assess riboregulatory activity [25]. We observed that mutations in the toehold completely abolished the activity of circRAJ31 (Figure 3(c)). Therefore, these results confirm that the exposed toehold in circRAJ31 triggers the activation of the *cis*-repressed sfGFP.

Figure 3Characterization of activation of gene expression by the engineered circular riboregulator. (a) Scheme of the construction used: the riboregulator circRAJ31 can *trans*-activate a *cis*-repressed gene coding for sfGFP with a degradation tag (LAA). IPTG induces production of the mRNA and aTc of the ribozyme RNA, which releases the circular riboregulator after self-splicing. (b) Scheme of the control systems constructed: the toehold mutant (to inactivate interaction), the C873U mutant (in intron 2, to inactivate self-splicing), and the linear riboregulator RAJ31min (without the T4-PIE ribozyme scaffold). (c) Characterization of systems based on circRAJ31, the native system, and its controls. See also the effect of different concentrations of IPTG and aTc in Figure S5. (d) Scheme of the construction used: the riboregulator circRAJ31 can *trans*-activate a *cis*-repressed *cat* gene, which codes for chloramphenicol resistance (CamR). (e) Characterization of circRAJ31 controlling growth of *E. coli* on chloramphenicol plates (35 *μ*g/mL), with appropriate inducers of gene expression (see the plates in Figure S7). (f) Characterization of circRAJ31 and the C873U mutant controlling growth of *E. coli* on liquid cultures (70 *μ*g/mL chloramphenicol), with appropriate inducers of gene expression (this plot is extended in Figure S8). In all cases, error bars indicate standard deviations.

**(a):**
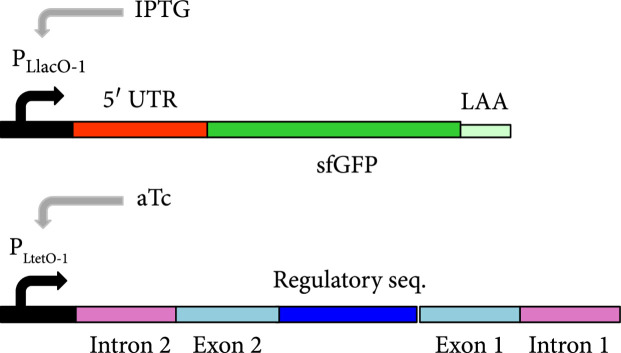

**(b):**
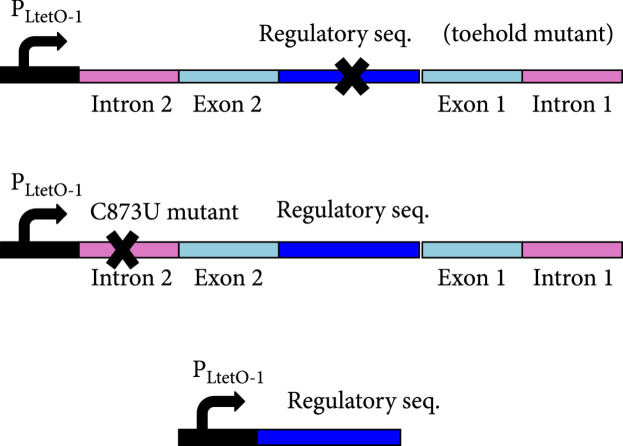

**(c):**
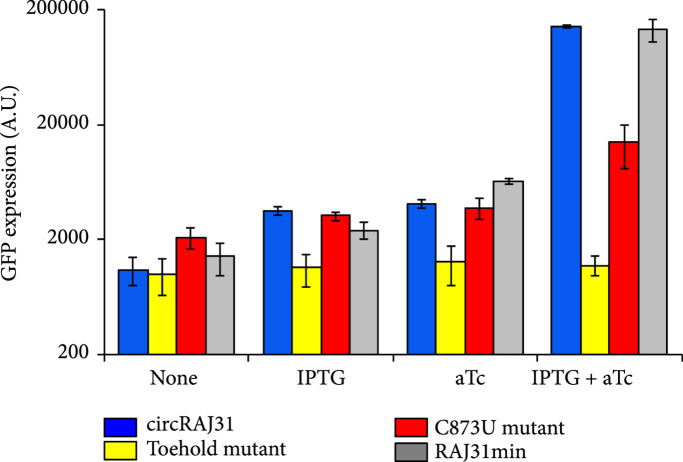

**(d):**
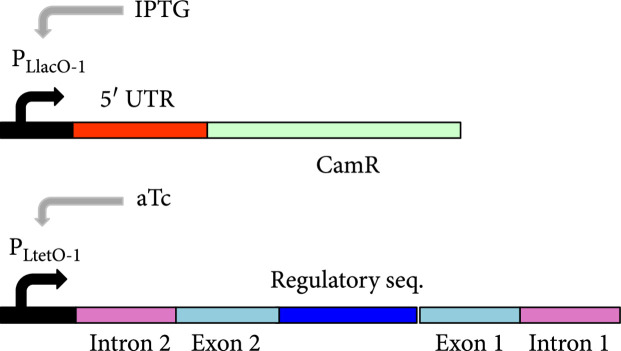

**(e):**
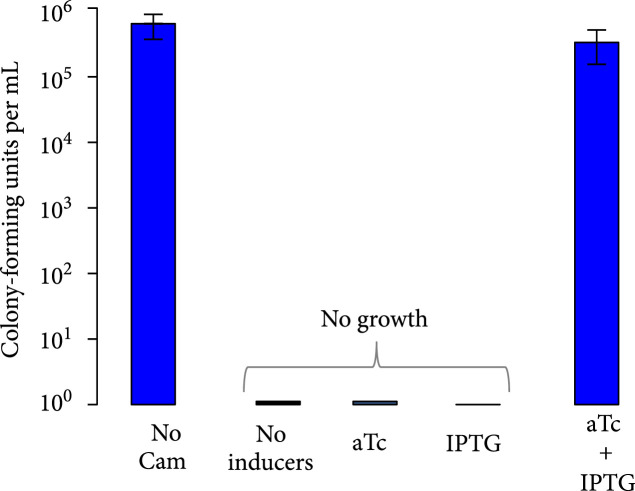

**(f):**
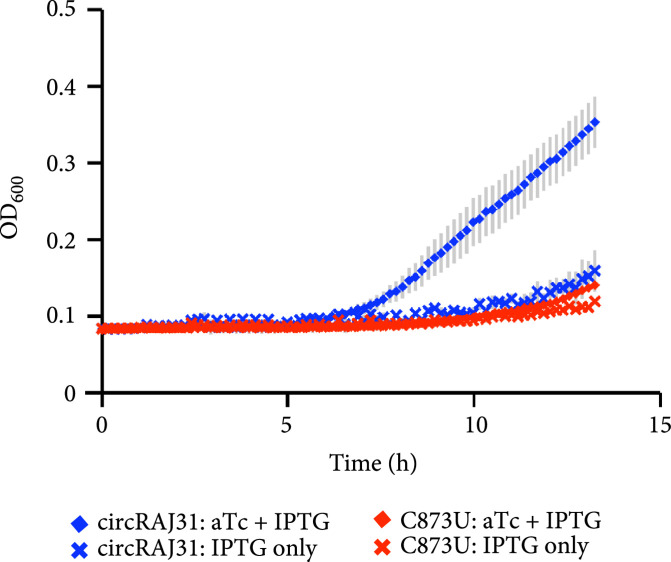

Because the T4-PIE ribozyme generates a heterogeneous population of circular and linear sRNAs, we decided to investigate if the unspliced version of the sRNA could still activate gene expression. For that, we constructed a mutant (C873U) known to have a very low efficiency of self-splicing (1.5% of wild type) [26, 27]. The mutation occurs in the P7 loop of the T4 ribozyme (intron 2 in our design; Figure 3(b)), and it contributes to the destabilization of the tertiary structure hence impinging on circularization. Using the same strategy as before with convergent and divergent primers (Figure 2(b)), the RT-PCR showed in this case a very faint band corresponding to the circular product (note also that the unspliced product was detected; Figure 2(c)). As before, RNA was purified from *E. coli* cells expressing this splicing-deficient mutant. However, *in vitro* transcription of this system showed that significant amounts of circular sRNA, as well as aberrant splicing products, are produced (Figures S3 and S4; note that the native system circRAJ31 circularized efficiently *in vitro*). Such dysfunctionality is known to occur when ribozymes are destabilized and then adopt different conformational states (i.e., misfolding) [28, 29]. In terms of activity, whilst the native system approximately led to a 40-fold increase in fluorescence, the observed fold change for the C873U mutant was around 4. A second splicing mutant (G423A, also in intron 2) gave similar results (Figure S6). We hypothesized that the large genetic context of the riboregulator (introns and exons) would be detrimental for function (i.e., interaction ability with the mRNA), as undesired base pair interactions are likely to occur (especially those blocking the toehold) [1, 23]. If so, a system expressing the sRNA RAJ31min not encased in a ribozyme would perform better than the C873U mutant. To confirm this hypothesis, we constructed this new system (Figure 3(b)), revealing a higher activation of sfGFP by the linear sRNA out of the ribozyme (Figure 3(c); expression levels comparable to those obtained with the circular sRNA).

### 3.3. Modeling RNA Processing and Regulation

To better understand the dynamical behavior of the system, we constructed a simple mathematical model. By denoting R, L, and C, the intracellular concentrations of the different sRNA species of the system (unspliced form, linear spliced form, and circular form, respectively), we wrote

(1)dRdt=α−βR−δR+μR,dLdt=βR−γL−δL+μL,dCdt=γL−δC+μC,

where α is the transcription rate, β is the intron splicing rate, and γ is the exon ligation rate. According to previous time course studies, the mean time for intron splicing (1/β) is about 20 min [17] (measured for a PIE ribozyme derived from *Anabaena*), and the mean time for exon ligation (1/γ) is about 15 min [30] (measured for a group I ribozyme from *Tetrahymena*). In addition, $\delta_{R}$, $\delta_{L}$, and $\delta_{C}$ are the respective degradation rates of the sRNAs, and μ is the cell growth rate. To quantify RNA processing, we here expressed the ribozyme with a T7 promoter in *E. coli* BL21(DE3) cells, which had a doubling time (1/μ) of about 30 min. By setting that the total concentration of sRNA is $R_{\text{tot}}=R+L+C$, we determined the fractions of each species in a steady state, i.e., $f_{R}=R/R_{\text{tot}}=0.72$, $f_{L}=L/R_{\text{tot}}=0.10$, and $f_{C}=C/R_{\text{tot}}=0.18$. These data allowed us, following the model, to estimate the degradation rates of the different species of the system. Note that we neglected in the model the partly spliced forms, which were nevertheless quantified together with the unspliced form. First, we obtained $\gamma f_{L}=\left(\delta_{C}+\mu \right)\;f_{C}$, which leads to a circular form with increased stability ($\delta_{C}\approx 0$), in agreement with previous results [14]. Notably, when $\delta_{C}\ll \mu$, the precise value of *δ_C_* is not a determinant of performance (from a mathematical perspective); atop, it is difficult to estimate from *in vivo* concentrations. Indeed, the growth rate imposes an effective degradation for all macromolecules in the cell. Second, we obtained $\beta f_{R}=\left(\gamma +\delta_{L}+\mu \right)\;f_{L}$, which gives a half-life of the linear spliced form of $1/\delta_{L}\approx 4\;\min$. Third, we roughly derived $f_{R}=\left(\delta_{R}+\mu \right)/\left(\beta +\delta_{R}+\mu \right)$, which gives a half-life of the unspliced form of $1/\delta_{R}\approx 10\;\min$.

Each of the sRNAs in the heterogeneous population will activate sfGFP in a different manner. By denoting g, the fold change in sfGFP expression due to the riboregulator, and by knowing that g scales linearly with the concentration of the riboregulator [31], we wrote

(2)g=1+σRfR+σLfL+σCfC,

where $\sigma_{R}$, $\sigma_{L}$, and $\sigma_{C}$ are the respective activation capacities of the sRNAs. In this case, we expressed the ribozyme with the P_LtetO1_ promoter in *E. coli* DH5*α*Z1 cells, assuming that the sRNA fractions were maintained. We measured g≈4 for the C873U mutant. With $f_{R}\approx 1$ and $f_{L}\approx f_{C}\approx 0$ in this case, we obtained $\sigma_{R}\approx 3$. Then, we estimated the value of $\sigma_{L}$ with the activity of the linear sRNA out of the ribozyme, g≈40 (note that this value is similar in the case of the native system). Because the half-life of the ribozyme is 2.5 times greater than the half-life of the linear form ($\delta_{L}/\delta_{R}\approx 2.5$, as previously estimated), we now needed to state $f_{L}\approx 0.4$ and $f_{R}=f_{C}=0$. Indeed, $f_{L}\approx 0.4$ is an upper bound, as 5${}^{\prime }$ cleaved RNAs tend to be more stable [32]. Accordingly, we obtained $\sigma_{L}\approx 100$. Finally, with the values of $f_{R}=0.72$, $f_{L}=0.10$, and $f_{C}=0.28$ for the native system, we got $\sigma_{C}\approx 140$. This suggests that the circular form is as functional as the linear form and that circularity is not a topological constraint in this framework. Of course, as the values of β and γ used here to estimate the rest of the parameter values do not correspond to *E. coli* (a bacterium that has a physiological context different from *Anabaena* and *Tetrahymena*), a certain degree of speculation might be attributed to our calculations. However, irrespective to the parameterization, this model is useful to describe the mode of action of the engineered riboregulatory system.

### 3.4. Controlling Cell Physiology with a Circular Riboregulator

We then tested the ability of circRAJ31 to control a selectable phenotype. To this end, we used an antibiotic resistance gene (the *cat* gene, which confers chloramphenicol resistance; Figure 3(d)). For a tight control, we would need an efficient repression of *cat* expression in the absence of the riboregulator (Figure S2 shows the predicted effect of different coding sequences on the secondary structure of the 5${}^{\prime }$ UTR), together with an efficient activation in its presence. We found that circRAJ31 is able to control the growth rate, resulting in growth of colonies on plates containing IPTG and aTc, but not on IPTG or aTc alone, after 20 h (Figure 3(e); see also Figure S7 where we show the corresponding plates). In liquid cultures, the presence of the riboregulator results in a faster growth rate and shorter lag phase at high concentration (70 *μ*g/mL) of chloramphenicol (Figure 3(f); see also Figure S8 for an extension in time and growing conditions). In the absence of aTc (i.e., no riboregulator), the bacterial population starts growing about 40% slower and with a delay of 6 h with respect to a culture with aTc, presumably due to leaky expression of the *cat* gene. This dynamics was also observed for the C873U mutant, as expected according to the fluorescence data. For lower concentrations (17.5 and 35 *μ*g/mL) of chloramphenicol, the differences between growth curves of systems expressing or not the riboregulator were reduced (Figure S9). Thus, circRAJ31 activates the antibiotic resistance to control the population growth, although a tighter control could be achieved with a stronger *cis*-repression.

## 4. Discussion

In this work, we have shown that a functional circular regulatory RNA can be engineered in *E. coli* cells. We based our system on a synthetic riboregulator previously designed by a computational approach (RAJ31) [19], which we encased within the T4-PIE ribozyme scaffold. This system self-splices to remove the introns and ligate the exons. We have revealed that the system indeed produces a circular sRNA *in vivo* (circRAJ31) and that this circular molecule is capable, by directly interacting with the corresponding mRNA, of activating gene expression of a fluorescent reporter and of an antibiotic resistance gene (controlling bacterial growth [10]). As the splicing is not entirely effective, the T4-PIE ribozyme generates a heterogeneous population of circular and linear sRNAs [17, 33]. One alternative to the T4-PIE system to engineer superior riboregulators would be the *Anabaena*-PIE system, which has recently been shown to display an increased splicing efficiency (about 90%) [34]. Another alternative would be the use of twister ribozymes to exploit the endogenous machinery for RNA circularization [35] or even the use of a viroid scaffold, although at the expense of coexpressing a suitable RNA ligase [36]. Furthermore, by using splicing-deficient mutants, we revealed that the unspliced sRNA performed worse, presumably because the large ribozyme scaffold hinders an efficient sRNA-mRNA interaction [1, 23]. In this sense, the use of T4-PIE circularization would allow the insulation of a given sRNA from its genetic context (e.g., to remove terminator sequences or transcribed portions of promoters [37]) to increase modularity.

The engineering of a novel type of sRNA part nevertheless raises some questions that will need to be explored in the future. These include how circularity influences the interaction of the sRNA with cellular proteins, such as Hfq [38, 39], which assists some sRNAs in their function, or such as RNA nucleases of the host [32, 40], all known to be (partly) dependent of 5${}^{\prime }$/3${}^{\prime }$ ends. They also include how the insertion of a given riboregulator sequence affects splicing efficiency, an aspect of importance when scaling up the design process. In addition to translation regulation, circular sRNAs might also be designed to decoy natural sRNAs in bacteria or even to interact with proteins [41], mimicking the mechanisms evolved in higher eukaryotes. Finally, as our ability to engineer regulatory RNAs able to work in *cis* (e.g., riboswitches [42]) or in *trans* increases, novel gene expression programs will be developed for synthetic biology applications.

## Supplementary Materials

Supplementary materials Figure S1: predicted secondary structure of circRAJ31. Figure S2: analysis of the secondary structure of the 5${}^{\prime }$ UTR and the first 30 bp of the coding sequence of gfp or cat. Figure S3: electrophoretic assay and sequencing results of RT-PCR after in vitro transcription. Figure S4: putative sequence of the RT-PCR misprocessing reaction. Figure S5: response of the system to varying concentrations of aTc (which controls expression of the riboregulator). Figure S6: characterization of mutant systems. Figure S7: growth of cells cotransformed with plasmids expressing circRAJ31 and CamR on plates without chloramphenicol (Cam) and on plates with chloramphenicol and different inducers. Figure S8: growth curves with a *cis*-repressed gene coding for chloramphenicol acetyltransferase (CamR). Figure S9: additional growth curves for different concentrations of Cam. Table S2: sequences of the primers used in this work.
