# Supplementary material for: Engineering a Circular Riboregulator in Escherichia coli
Source: Biodes Res. 2020 Sep 12;2020:1916789. doi: 10.34133/2020/1916789 (PMC10521646; doi:10.34133/2020/1916789)
Supplement: Supplementary materials — Figure S1: predicted secondary structure of circRAJ31. Figure S2: analysis of the secondary structure of the 5′ UTR and the first 30 bp of the coding sequence of gfp or cat. Figure S3: electrophoretic assay and sequencing results of RT-PCR after in vitro transcription. Figure S4: putative sequence of the RT-PCR misprocessing reaction. Figure S5: response of the system to varying concentrations of aTc (which controls expression of the riboregulator). Figure S6: characterization of mutant systems. Figure S7: growth of cells cotransformed with plasmids expressing circRAJ31 and CamR on plates without chloramphenicol (Cam) and on plates with chloramphenicol and different inducers. Figure S8: growth curves with a cis-repressed gene coding for chloramphenicol acetyltransferase (CamR). Figure S9: additional growth curves for different concentrations of Cam. Table S2: sequences of the primers used in this work. [file 1916789.f1.pdf]

Supporting Information

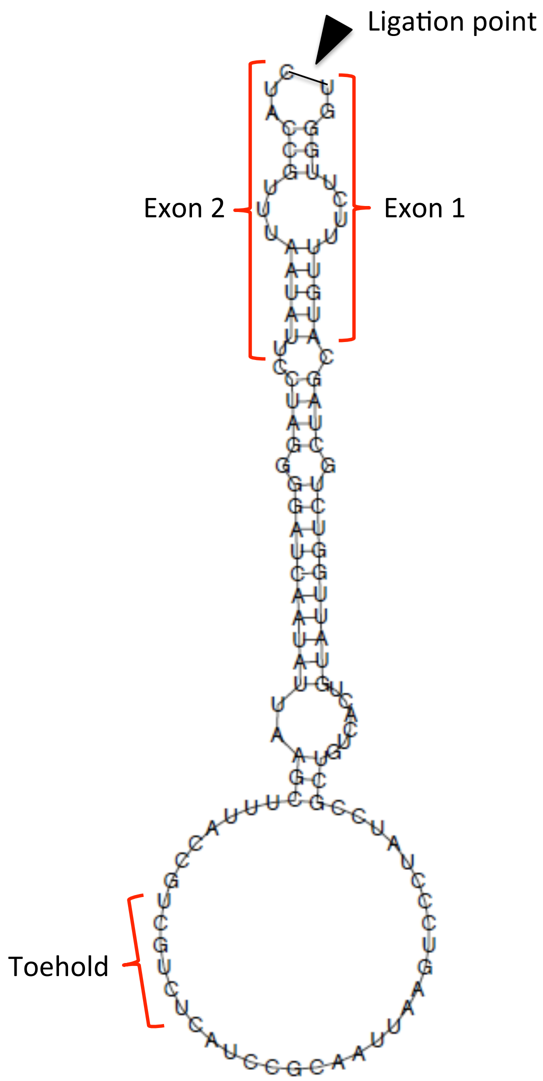

Figure S1. Predicted secondary structure of circRAJ31 with ViennaRNA webserver [1]. The toehold is exposed to allow interaction with the cognate 5' UTR. This circular riboregulator is based on the PIE ribozyme [2], created by circular permutation of the original T4 *td* ribozyme. The riboregulator is inserted between the two exons, and the sequence starts with the truncated P6 stem. Splicing of PIE ribozymes produces two linear RNAs composed of the introns and a circularized RNA that contains the two exons as well as inserted cargo (in this case, the RAJ31min riboregulator).

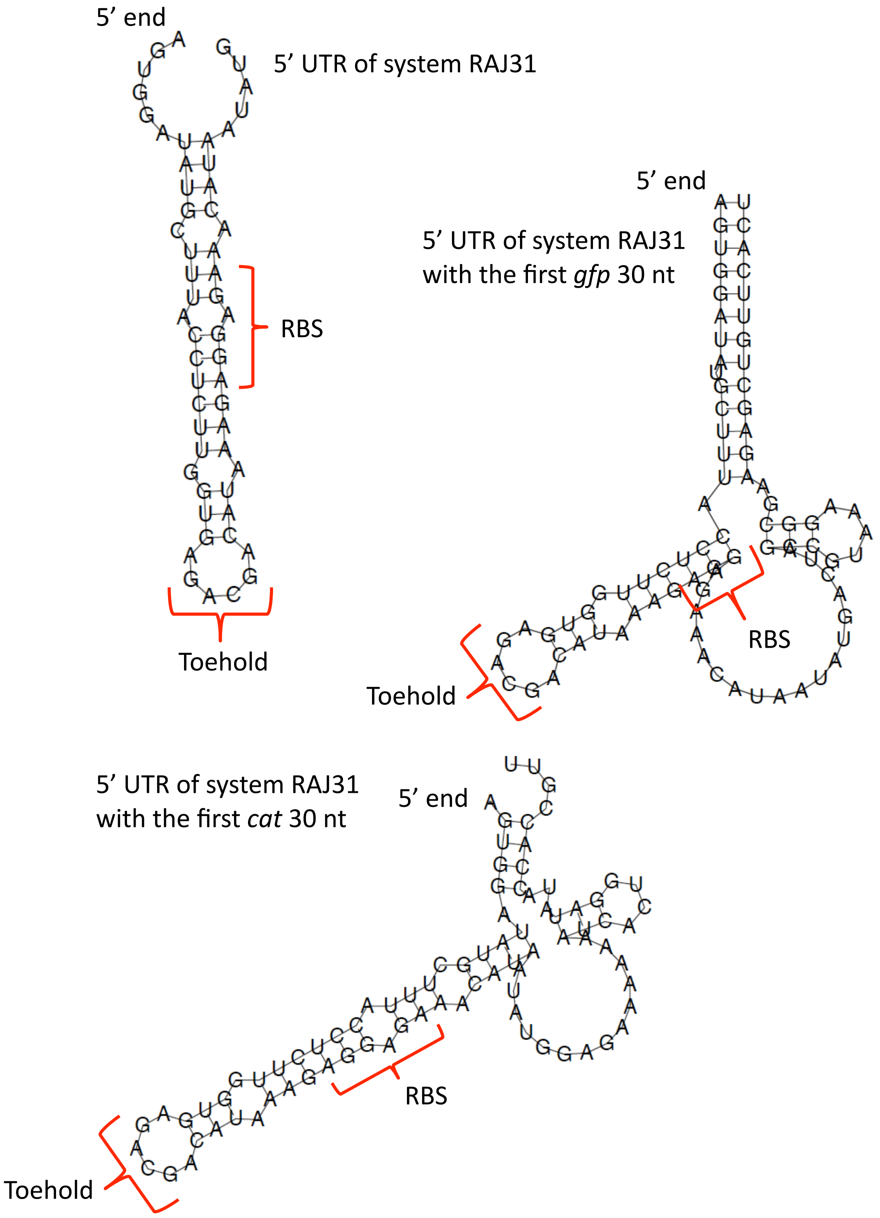

Figure S2. Analysis with ViennaRNA webserver [1] of the secondary structure of the 5’ UTR and the first 30 bp of the coding sequence of *gfp* or *cat*. The structures show that *gfp* gene partially affects the *cis*-repression of the RBS (which could explain some leakiness of the system in absence of the sRNA). The *cat* gene does not appear to interfere with the *cis*-repressing 5’ UTR to a significant extent.

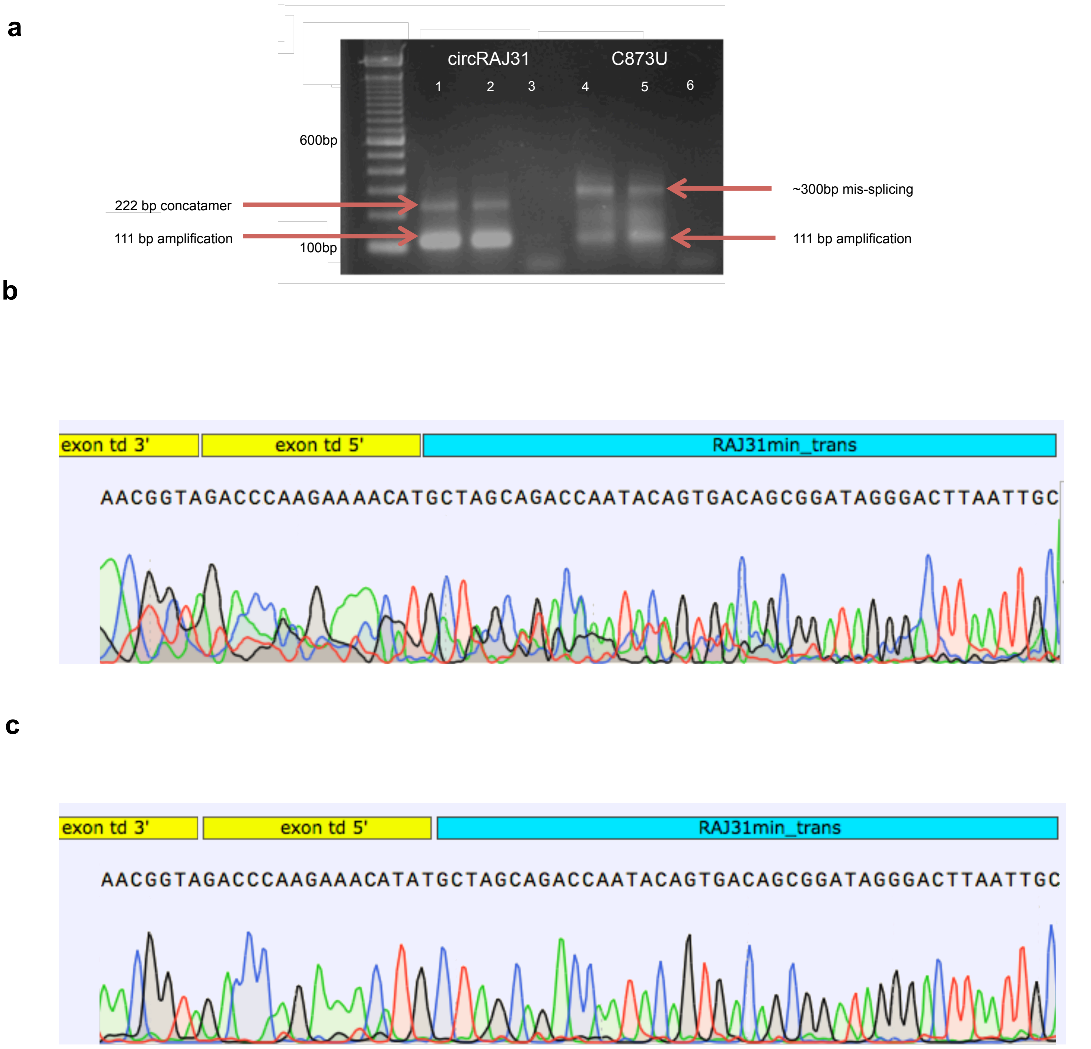

Figure S3. a) Gel of RT-PCR after *in vitro* transcription, amplified using primers CircRT-Fw (labelled A in fig. 2) and CircRT-Rv (labelled B in fig. 2). Lane 1: Invitrogen 100 bp ladder. Lane 2 and 3: Duplicates of the RT reaction with circRAJ31. Lane 4: No RT control. Lane 5 and 6: Duplicates of the RT with the C873U mutant. Lane 7: No RT. The RT-PCR of the circRAJ31 yields a 111 bp band (the length of the circular molecule, i.e., the length of RAJ31min and the two exons) and multiples of 111. The C873U mutant is known to have very low splicing efficiency (<1.5% of wild type [3]), and is still detected by the PCR (note that this is not the case with RNA produced *in vivo*). The ~300 bp band in the C873U lanes is a detected mis-splicing event, due to the destabilization of the normal ribozyme function by the C873U mutation (see fig. S4). This mis-splicing is only

detected in the *in vitro* transcription, but not on RT-PCR of RNA extracted from *E. coli* cells. b) Sequencing chromatogram of the RT-PCR product from a circRAJ31 *in vitro* transcription. c) Sequencing chromatogram of the RT-PCR product from a circRAJ31 produced *in vivo* in *E. coli.*

a

GGATGAGACGACGGTAAAGCTTAATATTGATCCCCTAGGaatattaaacggtagCATTATGTTCAGATAAGGTCGTTAATCTTACC
CCGGAATTATATCCAGCTGCATGTCACCATGCAGAGCAGACTATATCTCCAACTTGTTAAAGCAAGTTGTCTATCGTTTCGAGT
CACTTGACCCTACTCCCCAAAGGGATAATCGTTAGGCATTTATGTAGAACCTCTAGAacccaagaaaacatGCTAGCAGACCAATA
CAGTGACAGCGGATAGGGACTTAATTGC

b

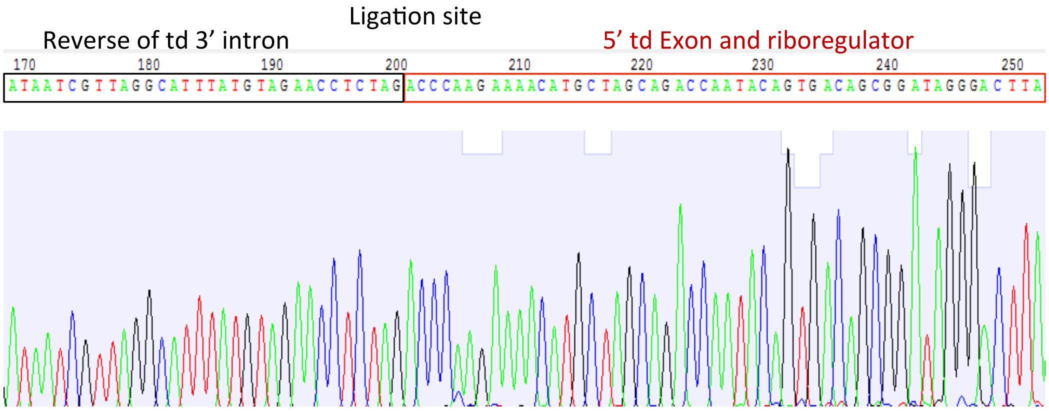

Figure S4. a) Putative sequence of the RT-PCR misprocessing reaction, where the 5’ exon (exon 1) is ligated to the 5’ of the RNA (intron 2, see also fig. S3), yielding a 284 bp circular RNA containing one intron. Sequence highlighting shows: RAJ31min sequence (cyan) starting with CircRT-Rv (primer B in fig. 2), exon (yellow), intron (pink), exon (yellow), and the other half of the RAJ31min sequence (cyan) ending with CircRT-Fw (primer A in fig. 2). Activation of the cryptic splice site yields some circular sequences that contain half of the intron, and are known to happen at low frequency when the splicing at the normal splice site are disturbed [4,5]. The mis-splicing resulted in the

ligation of the 5' exon (exon 1) to the start of intron 2 (at the 5' end of the PIE ribozyme RNA). Such splicing would yield a 284 bp band. Sequencing shows the removal of the GGG introduced by the T7 polymerase, and of the last U of exon 1. b) Chromatogram of the sequencing of circRAJ31-C873U RT-PCR product (bottom) around the ligation site and annotated sequence of the mis-spliced RNA (top). This mis-splice reaction was only detected after *in vitro* transcription, but not in samples produced in *E. coli.* However, it is possible that small amounts of aberrant products would also be produced *in vivo.*

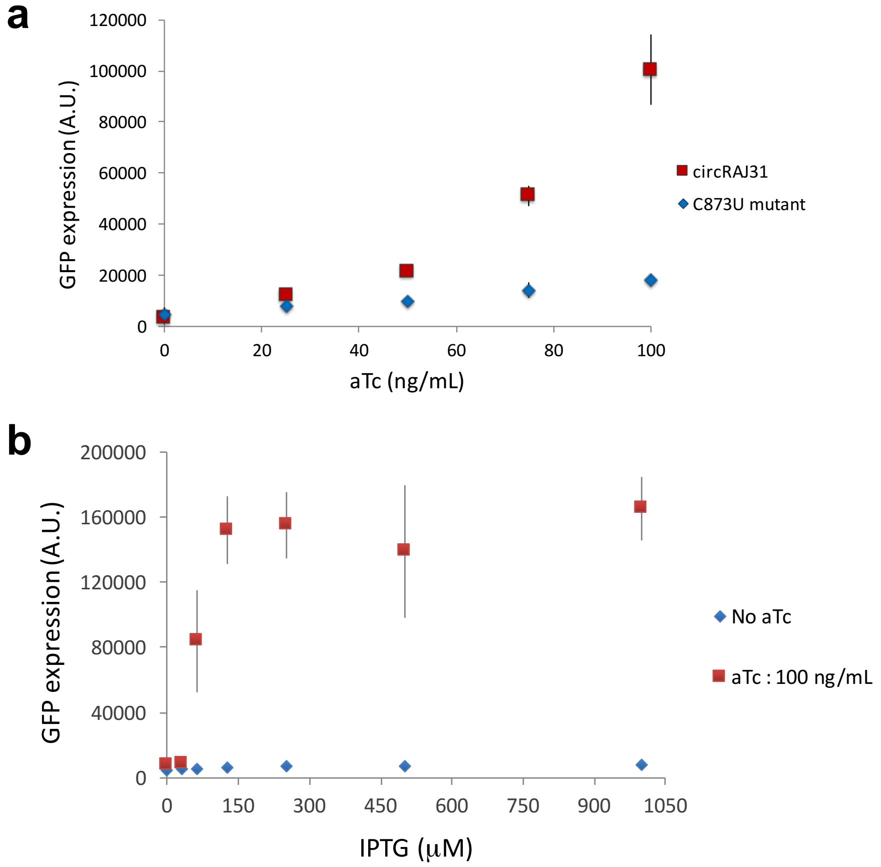

Figure S5. a) Response of circRAJ31 and C873U mutant to varying concentrations of aTc (which controls expression of the riboregulator), with constant IPTG 0.25 mM (which controls expression of the *cis*-repressed sfGFP mRNA). b) Response of circRAJ31 to varying concentrations of IPTG (which controls expression of the *cis*-repressed sfGFP

mRNA), with two constant levels of aTc, 0 and 100 ng/mL (which controls expression of the riboregulator). In these cases, the time points for analysis were closest to OD_{600} = 0.35 and auto-fluorescence of plain cells was not subtracted.

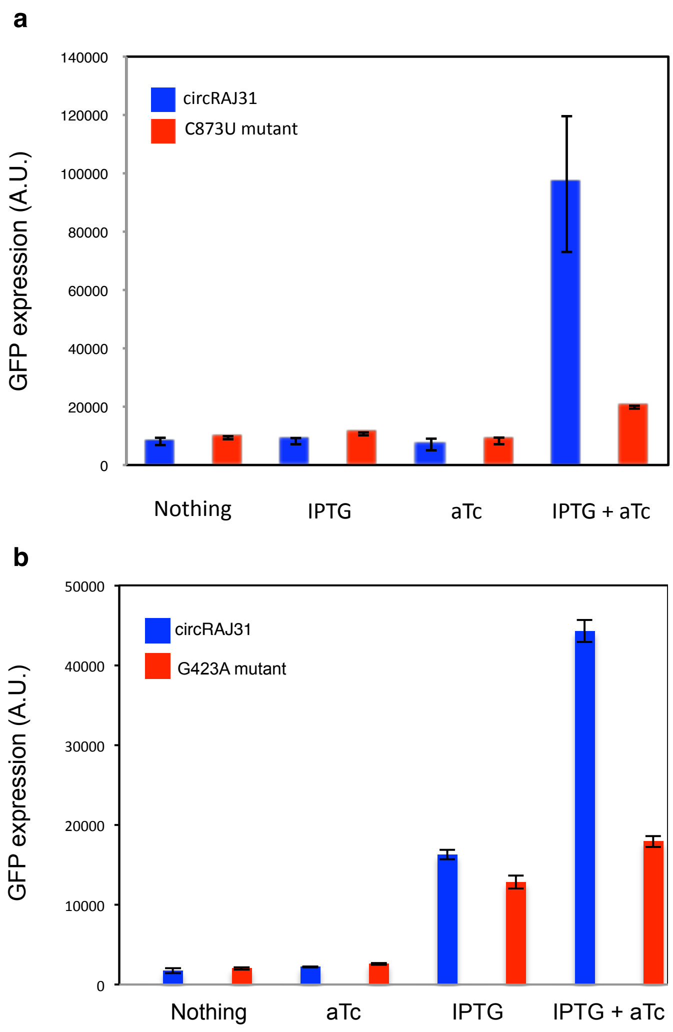

Figure S6. Characterization of circRAJ31 and a) the C873U mutant (IPTG 0.25 mM) and b) G423A mutant (IPTG 1 mM). In both cases (aTc 100 ng/mL), the reporter is a superfolder green fluorescent protein (sfGFP) without degradation tag. In this case, the time points for analysis were closest to OD_{600} = 0.35 and auto-fluorescence of plain cells was not subtracted.

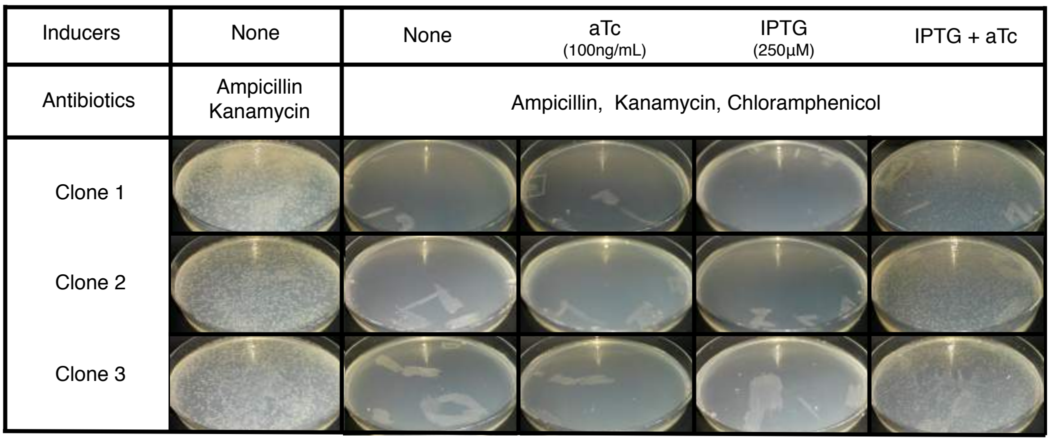

Figure S7. Growth of cells co-transformed with plasmids expressing circRAJ31 and CamR on plates without chloramphenicol (Cam), and on plates with chloramphenicol and different inducers.

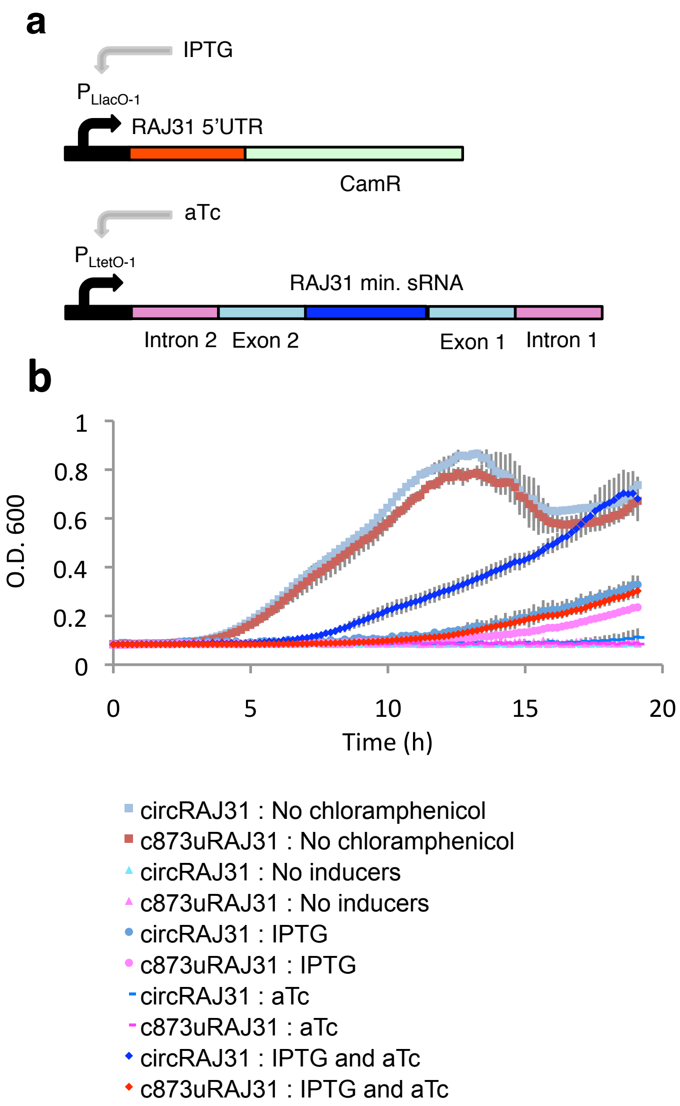

Figure S8. a) Scheme of the constructions used: riboregulator circRAJ31 can *trans*-activate a *cis*-repressed gene coding for chloramphenicol acetyltransferase (CamR). b) Growth curves (extension of fig. 3F with additional inducer conditions) of cells co-transformed with *cis*-repressed *cat* and either circRAJ31 or C873U mutant (the splicing deficient control) in LB broth, with or without chloramphenicol, or with and different inducer combinations. IPTG induces production of the *cis*-repressed mRNA, aTc induces production of the ribozyme, which releases the riboregulator after splicing.

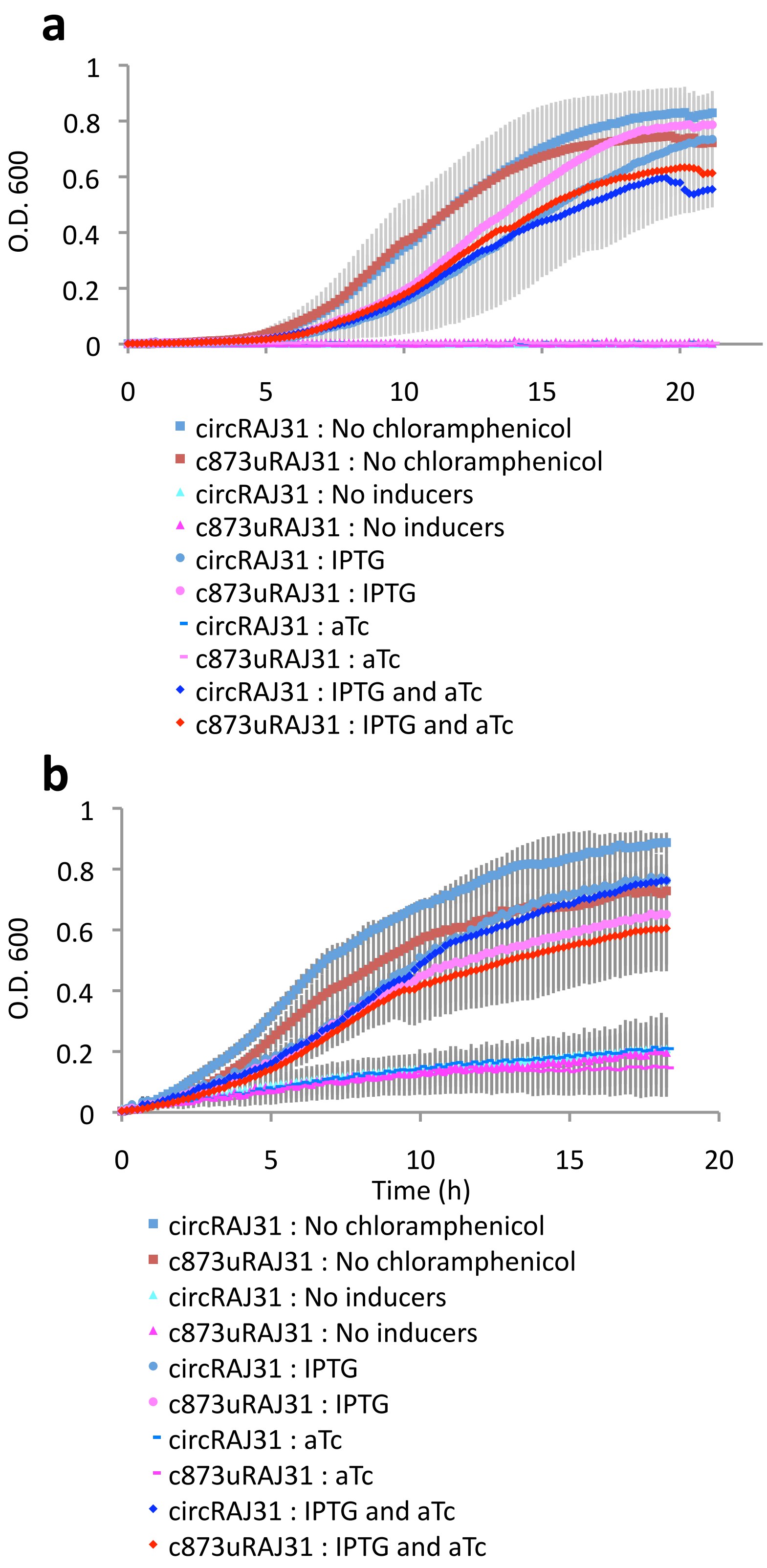

Figure S9: Additional growth curves of cells co-transformed with *cis*-repressed *cat* and circRAJ31 with a) 17.5 μg/mL chloramphenicol in presence or absence of inducers IPTG

(0.25 mM) and aTc (100 ng/mL), or b) 35 μg/mL chloramphenicol in presence or absence of inducers IPTG (0.25 mM) and aTc (100 ng/mL). Leakiness from the *cis*-repressed *cat* allows the cells to grow in the presence of IPTG alone, without activation by the riboregulator. At higher concentrations however, activation by the riboregulator significantly speeds up growth.

Table S1. Sequences of the system. To express circRAJ31, we used the P_{LtetO1} promoter and the *rrnC* terminator.

Name	Sequence
circRAJ31	GGTTCTACATAAATGCCTAACGACTATCCCTTTGGGGAGTAGGGTCAAGTGACTCGAAACGATAGACAACTTGCTTTAACAAGTTGGAGATATAGTCTGCTCTGCATGGTGACATGCAGCTGGATATAATTCCGGGGTAAGATTAACGACCTTATCTGAACATAATGctaccgtttaatattCCTAGG**GGATCAATATTAAGCTTTACCGTCGTCTCATCCGCAATTAAGTCCCTATCCGCTGTCACTGTATTGGTCT**GCTAGCatgttttcttgggtTAATTGAGGCCTGAGTATAAGGTGACTTATACTTGTAATCTATCTAAACGGGGAACCTCTCTAGTAGACAATCCCGTGCTAAATTGTAGGACT
Intron 2	GGTTCTACATAAATGCCTAACGACTATCCCTTTGGGGAGTAGGGTCAAGTGACTCGAAACGATAGACAACTTGCTTTAACAAGTTGGAGATATAGTCTGCTCTGCATGGTGACATGCAGCTGGATATAATTCCGGGGTAAGATTAACGACCTTATCTGAACATAATG
Exon 2	CTACCGTTTAATATT
Intron 1	TAATTGAGGCCTGAGTATAAGGTGACTTATACTTGTAATCTATCTAAACGGGGAACCTCTCTAGTAGACAATCCCGTGCTAAATTGTAGGACT
Exon 1	ATGTTTTCTTGGGT
C873U mutant in intron 2	GGTTCTACATAAATGCCTAACGATTATCCCTTTGGGGAGTAGGGTCAAGTGACTCGAAACGATAGACAACTTGCTTTAACAAGTTGGAGATATAGTCTGCTCTGCATGGTGACATGCAGCTGGATATAATTCCGGGGTAAGATTAACGACCTTATCTGAACATAATG
G423A mutant in intron 2	GGTTCTACATAAATGCCTAACGACTATCCCTTTGGGGAGTAGGGTCAAGTGACTCGAAACGATAGACAACTTGCTTTAACAAGTTGGAGATATAGTCTGCTCTGCATGGTGACATGCAGCTGGATATAATTCCGGGGTAAGATTAACGACCTTATCTGAACATAATA
Minimal version of sRNA RAJ31 flanked by two restriction sites	CCTAGGGGATCAATATTAAGCTTTACCGTCGTCTCATCCGCAATTAAGTCCCTATCCGCTGTCACTGTATTGGTCTGCTAGC (toehold highlighted)
Toehold mutant in RAJ31min	CCTAGGGGATCAATATTAAGCTTTACCGACACCCCATCCGCAATTAAGTCCCTATCCGCTGTCACTGTATTGGTCTGCTAGC
Cognate 5' UTR of sRNA RAJ31	AGTGGATATGCTTTACCTCTTGGTGAGACGACATAAAGAGGAGAAACATAATATG (RBS highlighted)

Table S2. Sequences of the primers used in this work.

Name	Sequence
tdC873U-Fw	ctacataaatgcctaacgaTtatccctttggggagtagg
tdC873U-Rv	cctactccccaaagggataatcgttaggcatttatgtag
g423a	GTAAGATTAACGACCTTATCTGAACATAATACTGTTTAATATTCCT
g423as	AGGAATATTAAACGGTAGTATTATGTTCAGATAAGGTCGTTAATCTTAC
CircRT-Fw	ggatgagacgacggTAAAGC
CircRT-Rv	gcaattaagtccctatccgCTG
CircRT-Lin	GACATGCAGCTGGATATAATTCC
Toe31-Fw	ttGGTCTCtGTCCCTATCCGCTGTCACTG
Toe31-Rv	aaGGTCTCaCCCCTAGGAATATTAAACGGTAGC

References

[1] Gruber, A.R., Lorenz, R., Bernhart, S.H., Neuböck, R., and Hofacker, I.L. The Vienna RNA Websuite. *Nucleic Acids Res.* 2008, 36, 70-74.

[2] Puttaraju, M., and Been, M.D. Group I permuted intron-exon (PIE) sequences self-splice to produce circular exons. *Nucleic Acids Res.* 1992, 20, 5357-5364.

[3] Brion, P., Michel, F., Schroeder, R., and Westhof, E. Analysis of the cooperative thermal unfolding of the td intron of bacteriophage T4, *Nucleic Acids Res*. 1999, 27, 2494-2502.

[4] Price, J.V., Engberg, J., and Cech, T.R. 5' exon requirement for self-splicing of the Tetrahymena thermophila pre-ribosomal RNA and identification of a cryptic 5' splice site in the 3' exon. *J. Mol. Biol.* 1987, 196, 49-60.

[5] Chandry, P.S., and Belfort, M. Activation of a cryptic 5’ splice site in the upstream exon of the phage T4 td transcript: exon context, missplicing, and mRNA deletion in a fidelity mutant. *Genes Dev*. 1987, 1, 1028-1037.
